# Resolution of Vascular Compromise From Liquid Rhinoplasty Using *Hirudo medicinalis* Therapy

**DOI:** 10.1093/asjof/ojae067

**Published:** 2024-08-23

**Authors:** Alexander Rivkin, Solomiia Chepka

## Abstract

Hyaluronic acid (HA) fillers are a relatively safe and effective means of cosmetic treatment for the face; however, as the numbers of both patients and injectors continue to rise, reports of adverse events (AEs) and ischemia are increasing. Although visual complications and stroke are the most-feared AEs, skin and underlying tissue necrosis is far more common and can be catastrophic. HA can be dissolved with hyaluronidase, but this does not always resolve ischemia. In some instances, including the case presented here, conventional interventions are inadequate to reverse the progression of ischemia and restore blood flow. In this case study, HA injection of the nasal sidewall resulted in ischemia and impending necrosis of the nasal tip. Following failure of standard-of-care measures to reverse the progression of ischemia and restore blood flow, *Hirudo medicinalis* therapy was successfully used as an adjuvant treatment. To our knowledge, this is the first report of *H. medicinalis* therapy for treatment of ischemia and necrosis from aesthetic filler injection. Based on experience here, this approach should be considered for patients who are out of therapeutic options, or as a helpful adjunct to speed resolution of vascular occlusion. In addition, the success of *H. medicinalis* therapy, which acts locally on the microvasculature, may inform our understanding of the mechanism of vascular occlusion with fillers.

**Level of Evidence: 5 (Therapeutic):**

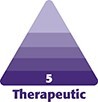

Hyaluronic acid (HA) fillers are a relatively safe and effective means of cosmetic treatment for the face. Their price point and lack of recovery time make aesthetic improvement accessible to a large number of people. In 2022, an estimated 4.3 million patients received HA filler injections worldwide,^1^ and their use continues to increase rapidly year after year. Adverse event (AE) rates with filler use are low; however, reports are increasing as the number of injectors, and the number of patients continues to rise. Although visual complications and stroke are the most-feared AEs, skin and underlying tissue necrosis are far more common and can be catastrophic.^2^ Currently, the overall risk of necrosis for facial filler injection is estimated to be about 0.001% to 0.01%^3,4^; however, this number is an estimate, and rates vary significantly based on the area being injected, as well as the experience, skill, and preparedness of the injector. One area of the face particularly prone to complications from filler injection is the nose, especially in patients with a history of prior surgical rhinoplasty.^5,6^

Here, we present a case of ischemia and impending necrosis of the nasal tip resulting from HA injection of the nasal sidewall in a patient with a history of multiple surgical rhinoplasty procedures. Conventional interventions were inadequate to reverse the progression of ischemia and restore blood flow, prompting the senior author to use *H. medicinalis* therapy as an adjuvant treatment. To our knowledge, this is the first report of *H. medicinalis* therapy for treatment of ischemia and necrosis from aesthetic filler injection.

## CASE PRESENTATION

A 33-year-old female with a current history of smoking presented expressing dissatisfaction with what she believed to be unnatural contours of her nose. She had undergone a closed surgical rhinoplasty 8 years prior, which was revised 3 years later. Three years after her revision surgery (2 years prior to the current filler injection), she underwent nonsurgical rhinoplasty (NSR). The NSR was complicated by ischemia of her nasal tip secondary to a vascular occlusion of her left sidewall; however, ischemia resolved after immediate treatment with hyaluronidase (HYAL) 150 units/mL (Hylenex Halozyme Therapeutics, San Diego, CA) and hyperbaric oxygen (HBO).

**Figure 1.**
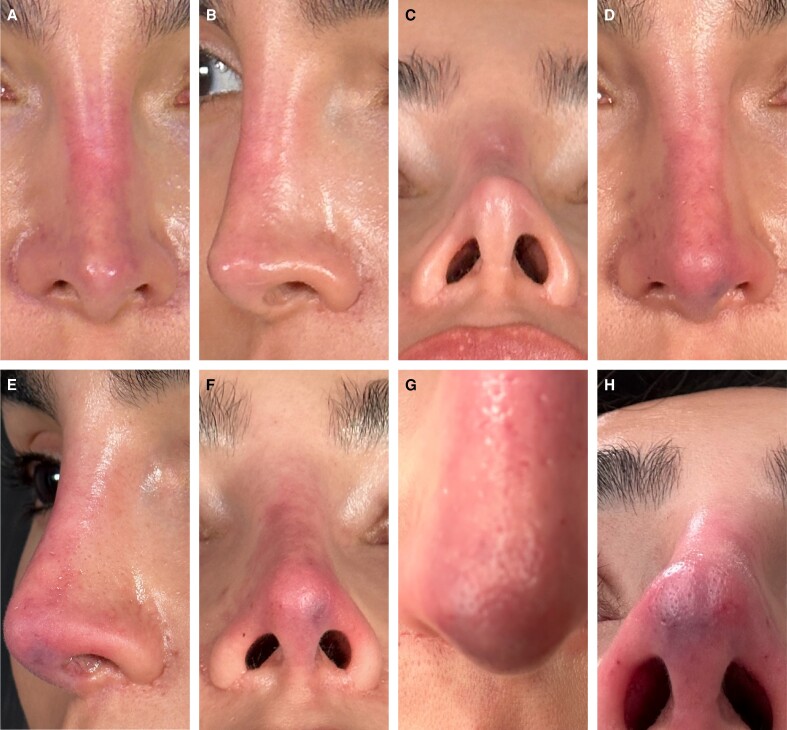
A 33-year-old female patient is shown at baseline, prior to undergoing nonsurgical rhinoplasty (NSR) and touch-up injection (A-C). The NSR described in this case was done 8 years following a surgical rhinoplasty, 3 years following a revision, and 2 years following her first NSR. During the touch-up injection (performed 2 months after the second NSR), just after injection in the right sidewall, the tip and right sidewall of the nose blanched and displayed delayed capillary refill. The patient is shown immediately following injection, after treatment with aspirin, hyaluronidase, nitroglycerin paste, methylprednisolone, and nitrous oxide gas. At this time, the infratip lobule remained dusky with slow capillary refill (D-F). Lastly, the patient is shown immediately after hyperbaric oxygen treatment (G and H), administered on the day of the injection. At this time, the duskiness was still not resolved.

Examination revealed dorsal asymmetry; an overly narrow supratip; and a pinched, overly sharp, and overrotated tip with collapse of the right alar cartilage (Figure 1A-C). Nasal skin capillary refill was normal. The patient was counseled that NSR carried significant risk of ischemia and necrosis, given her surgical, injectable, and smoking history. Because the patient had both reasonable expectations for the aesthetic outcome as well as a firm understanding of treatment risk, it was reasonable to proceed when the patient decided to undergo treatment. Furthermore, the risk, expense, and downtime of revision surgery would have been significantly greater, and unlikely to accomplish the patient's aesthetic goals. The goals of this revision NSR procedure were to improve dorsal symmetry by augmenting the right sidewall, to augment and widen the supratip, to derotate the tip by adding to the infratip lobule, to soften the tip contours, and to project the right ala laterally and inferiorly.

Prior to the procedure, the patient was given aspirin 325 mg, and a compounded topical numbing cream (lidocaine 23%, tetracaine 7%) was applied for 15 min. The HA filler, Vycross 20 mg/mL (VYC-20L; Juvéderm Voluma; Allergan Aesthetics, Madison, NJ), was back-loaded into a 0.3 cc Becton Dickinson syringe with a fixed 0.8 mm, 31 G needle in a sterile manner by removing the plunger and injecting 0.2 cc into the syringe using a 21 G, 1 inch needle on the VYC-20L syringe. A total of 0.35 cc was injected, and the patient reported satisfaction with the resulting nasal contours.

Two months later, the patient returned for a customary touch-up evaluation. Some of the previous filler had settled, and the patient wanted further tip derotation and correction of her dorsal asymmetry. The patient was injected with NASHA-L (Restylane Lyft; Galderma Laboratories, Fort Worth, TX). A total of 0.2 cc was used: 0.1 cc in the dorsum, 0.05 cc in the sidewall, and 0.05 cc in the infratip lobule. Immediately after the last injection in the right sidewall, the tip and right sidewall of the nose blanched and displayed delayed capillary refill. The patient did not complain of pain. Vigorous massage and hot compresses to the area for 25 min did not result in improvement, and the patient developed livedo reticularis over the dorsum, right sidewall, tip, and infratip lobule. An additional dose of aspirin 325 mg was given. The sidewall and tip were then injected with 300 units of HYAL over 2 sessions, 30 min apart; nitroglycerin ointment (Nitro-Bid, Savage Laboratories, Melville, NY) was applied every 15 min for 1 h (4 times); the first dose of a methylprednisolone dose pack (Medrol 12 mg; Pfizer, New York, NY) was given; and the patient was given nitrous oxide gas to inhale for 1 h.

Following the above-detailed steps, the sidewall and dorsum recovered normal blood flow; however, the infratip lobule remained dusky with slow capillary refill (Figure 1D-F). The patient was sent for urgent HBO treatment. Her duskiness was unresolved after HBO (Figure 1G, H), so the decision was made to send the patient for *H. medicinalis* treatment.

**Figure 2.**
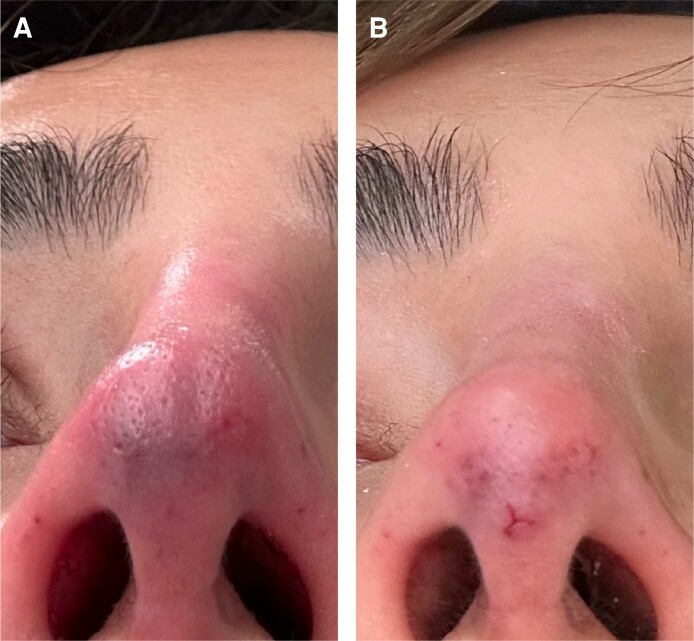
A 33-year-old female patient is shown (A) after treatment with aspirin, hyaluronidase, nitroglycerin paste, methylprednisolone, nitrous oxide gas, and hyperbaric oxygen and (B) immediately after a single *H. medicinalis* treatment of the infratip lobule for 45 min.

**Figure 3.**
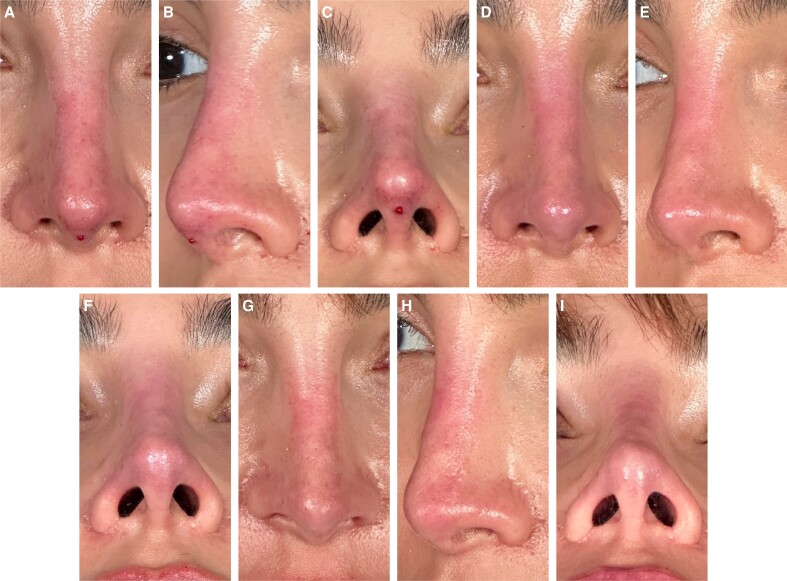
A 33-year-old female patient is shown 2 days following the injection, after one 45-min *H. medicinalis* treatment, followed by 2 more daily sessions of hyperbaric oxygen (A-C); the patient is shown a few hours after both treatments. The patient is also shown 12 days (D-F) and 30 days postinjection (G-I).

**Figure 4.**
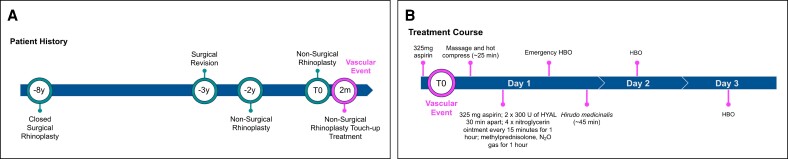
Timeline of the case study patient's history (A) and treatment for vascular compromise (B).

The patient was pretreated with a sulfonamide antibiotic, and a single leech was placed on her infratip lobule for 45 min (Video). Significant resolution was observed immediately after the treatment (Figure 2), with markedly improved skin color and capillary refill. All of the above treatments were accomplished within a single day. The patient received 2 more daily HBO treatments and is shown after the second HBO treatment, 2 days after the occlusion, in Figure 3A to C. Her skin remained healthy through her follow-up visits at Day 12 (Figure 3D-F) and Day 30 after injection (Figure 3G-I). A timeline showing the course of events and treatment is shown in Figure 4. Because this is not a study, Internal Review Board approval was not obtained. The patient was treated according to best clinical practices, provided informed consent for treatment, and provided written consent for publication of her photographs.

## DISCUSSION

All cosmetic injectors, whether physician, nurse, nurse practitioner, or physician's assistant, must be able to immediately recognize and effectively treat skin and soft-tissue ischemia. Aesthetic patients should be confident that any provider they see utilizes the latest preventive measures and knows exactly what to do in the unlikely case of an emergency. Recognizing ischemia should be straightforward. Blanching of the skin and slow capillary refill that does not recover after a couple of minutes of massage is pathognomonic, whether or not it is accompanied by unusual pain, especially if followed a few minutes later by dusky skin or a livedo reticularis pattern of mottled skin discoloration.^6^ Capable aesthetic injectors should be able to differentiate ischemia from ecchymosis by the appearance and pattern/area of occurrence. In the case above, the author knew from experience that the nasal tip rarely bruises: dark discoloration in the tip must be presumed to be ischemic and treated accordingly. In this case, the authors were confident that the discoloration was not a bruise because of how quickly the discoloration occurred, the dark color observed (which was less reminiscent of bruising and more like that of ischemia), the slow capillary refill, the fact that the area blanched first and then darkened, and the fact that there were multiple areas affected at once (both the sidewall, which resolved with measures aimed at vascular occlusion, and the tip). Reestablishing blood flow can prevent the progression of initial pallor and livedo reticularis (because of arterial insufficiency causing venous dilation and an increase in deoxygenated hemoglobin) to blistering, pustules (indicating breakdown of skin integrity and bacterial overgrowth), tissue necrosis, and eschar formation soon thereafter^3,4,7^ (usually within 3-6 days).

Ischemia from filler injection is usually caused by intravascular filler occlusion or spasm, although compression and compartment effect can be a factor in tight areas like the nasal tip. Hyaluronic acid is a potent vascular irritant, and many cases of ischemia, if identified immediately, can be resolved with massage and heat, which vasodilate and help resolve vasospasm. This prevents a possible feed-forward loop in which vasospasm causes ischemia, which in turn releases inflammatory factors that cause further vasospasm and ischemia.^8^ If the tissue does not completely recover normal color and capillary refill from massage and heat, second-tier treatments should be initiated, including aspirin, oral or intramuscular steroids, HYAL injection, topical nitroglycerin application, and inhaled nitrous oxide. If these measures are unsuccessful, the patient should be referred to HBO. It is important to note that although part of the appeal of HA fillers is their reversibility with HYAL, this is not always effective or sufficient to limit injury, so it is critical to be prepared to manage vascular compromise that is not resolved by HYAL.

*Hirudo medicinalis* has been used in medicine for millennia. In more recent years, leeches have been used successfully to treat vascular compromise because of venous congestion in local, pedicled, and free flaps^9^ and in replanted digits, ears,^10^ and lips,^11^ as well as for postrhinoplasty skin ischemia. In a recent metareview of 67 case studies, salvage rates were between 60% and 100%.^12^

Leech application helps resolve venous congestion through the activity of a number of salivary components, which the leech uses to prevent its host's blood from clotting so that it can be ingested. Hirudin, the best-known ingredient, is a powerful inhibitor of thrombin, making it a more potent anticoagulant than heparin and an effective antiplatelet agent.^13^ Other components include HYAL and collagenase, which increase tissue permeability to hirudin, a histamine-like vasodilator, which increases blood flow to the area, and various antiplatelet agents such as calin, aspyrase, and saratin.^13^ In the case presented here, these substances were able to restore blood flow following vascular compromise from HA filler injection when other standards of care had failed. It is our hope that *H. medicinalis* therapy will be considered for patients who are out of therapeutic options, or as a helpful adjunct that may speed resolution of vascular occlusion.

In addition to providing some evidence in support of *H. medicinalis* therapy for managing ischemia, this case also sheds some light on the mechanisms of ischemia and necrosis. Aside from vasospasm, most published literature subscribe to the hypothesis that an intravascular filler plug obstructs a vessel or major branch and cuts off blood flow to the corresponding angiosome. However, this case report provides evidence for the alternative view—that vascular occlusion may be more of a microvascular than macrovascular phenomenon. We know that the effect of leech saliva is localized to the peripheral microvasculature. The fact that the treatment worked so well suggests that the obstruction to blood flow in this case was at the level of the capillaries. If the obstruction were more central, the treatment would have probably failed. This hypothesis is consistent with the research published by Ugradar et al, which showed that both calcium hydroxyapatite and HA fillers become microparticles immediately after being injected because of the shear stress of systolic blood pressure.^14^ Their conclusion was that vascular occlusion probably occurs through microemboli obstructing a critical mass of capillaries within the affected angiosome rather than a column of filler blocking an artery. In this case, management of the peripheral vasculature was sufficient to resolve treatment-resistant ischemia. Here, because the patient had slow capillary refill, it is likely that the compromise was arterial in nature; however, at the level of the capillary bed, arterial insufficiency leads to venous stasis and both contribute to ischemia. Improving sluggish movement of blood through these vessels can mitigate the impact of the compromise on tissues. Re-establishing venous capillary drainage and dissolving any intraluminal clots is critical to optimal re-establishment of tissue perfusion. Furthermore, if the discoloration was simply a bruise, one would expect *H. medicinalis* therapy to worsen the appearance of the bruise.

This study provides some evidence for the use of *H. medicinalis* therapy; however, additional research is needed to define efficacy within the context of different types of vascular compromise. This study is limited in that it is a single case study. Furthermore, in the presented case, although the marked resolution immediately after *H. medicinalis* treatment suggests causation, it is difficult to be absolutely certain, considering several treatment modalities were used. In addition, given that most vascular occlusions are not detected until about 24 to 48 h after injection, when the patient calls the office complaining of bruising and/or pain,^2^ it will be important to determine the window of time in which *H. medicinalis* therapy is effective.

## CONCLUSIONS

In the case study presented here, *H. medicinalis* therapy may have effectively restored blood flow in the infratip lobule after it seemed that standard measures for resolving vascular occlusion had failed. To our knowledge, this is the first time this has been reported in the literature. This case study paves the way for future research in this area, provides an avenue of treatment for clinicians and patients in complex cases of tissue ischemia, and may inform our understanding of the mechanism of vascular occlusion with fillers.
